# Determination of the effects of pulmonary arterial hypertension and therapy on the cardiovascular system of rats by impedance cardiography

**DOI:** 10.3325/cmj.2014.55.498

**Published:** 2014-10

**Authors:** Belgin Buyukakilli, Serkan Gurgul, Derya Cıtırık, Olgu Hallioglu, Murat Ozeren, Bahar Tasdelen

**Affiliations:** 1 Department of Biophysics, Faculty of Medicine, Mersin University, Mersin, Turkey; 2 Department of Biophysics, Faculty of Medicine, Gaziosmanpasa University, Tokat, Turkey; 3 Department of Pediatric Cardiology, Faculty of Medicine, Mersin University, Mersin, Turkey; 4 Department of Cardiovascular Surgery, Faculty of Medicine, Mersin University, Mersin, Turkey; 5 Department of Biostatistics, Faculty of Medicine, Mersin University, Mersin, Turkey

## Abstract

**Aim:**

To evaluate the effects of bosentan, sildenafil, and combined therapy on the cardiovascular system using impedance cardiography (ICG) in rats with monocrotaline (MCT)-induced pulmonary arterial hypertension (PAH).

**Methods:**

Seventy male Wistar-albino rats were randomized into five groups. A single dose of MCT was given to all rats, except to the control group. After 4 weeks, bosentan, sildenafil, and combined treatment was started and lasted for 3 weeks. The last group that developed PAH did not receive any medication. Echocardiographic evaluation was performed to determine the PAH development. Thoracic fluid content index (TFCI), stroke volume index (SI), heart rate (HR), cardiac index (CI), and myocardial contractility index (IC) were determined. All procedures were performed at the baseline and after 4 and 7 weeks.

**Results:**

Echocardiographic parameters showed that the all MCT-injected rats developed PAH. There were no significant inter- and intra-group differences in TFCI, SI, and IC (*P* > 0.05), but at the 7th week, CI value in the sildenafil-treated PAH rats was significantly higher than in other groups and HR of PAH rats with combined therapy was significantly lower than in other groups.

**Conclusion:**

PAH did not have an effect on LV function of rats, or if it did, the effect was compensated by physiological processes. Also, sildenafil treatment deteriorated the LV cardiac index.

Pulmonary arterial hypertension (PAH) is a chronic lung disease characterized by increased pulmonary artery pressure, pulmonary vascular damage, and medial hypertrophy of pulmonary arterioles, leading to right ventricular (RV) hypertrophy, RV failure, and eventually death (1). The monocrotaline (MCT)-induced model of PAH is the most used model in rats. MCT is a pyrrolizidine alkaloid from the plant *Crotalaria spectabilis*. A single injection of MCT results in injury to the vascular endothelium of the lung, pulmonary hypertension, and RV hypertrophy and failure within 3 or 4 weeks (2-5).

A key feature of PAH is deregulation of important vasodilatory mechanisms in the pulmonary circulation, including increased expression of phosphodiesterase 5 (PDE5) (6). Acute and chronic experimental models of PAH use PDE5 inhibitor sildenafil to reduce pulmonary pressure (7,8). Another important PAH treatment method is by endothelin (ET) receptor antagonist bosentan (9). The endothelin system is highly active in PAH and causes sustained vasoconstriction of pulmonary arteries. It increases the autogenic activity of smooth muscle cells and fibroblasts in the pulmonary vessel wall, thereby decreasing the lumen of pulmonary vessels and also contributing to increased pulmonary vascular resistance (10,11). It is also known that ET receptor expression in the RV myocardium increases due to PAH (12).

Heart failure due to PAH can be identified by clinical symptoms that are linked to hemodynamic indices of right heart failure (3,13). Because of the major differences between left and right heart hemodynamics and potentially different responses to pressure overload, the data obtained for RV myocardium do not have to be applicable to the left ventricle (LV). In addition, although chronic pulmonary hypertension selectively overloads the RV, there also manifests LV dysfunction (14). However, hemodynamic indices of the left heart and treatment effects on them cannot be identified in this situation. The aim of this study is to evaluate the effects of PAH and bosentan, sildenafil, and combined treatments on the LV hemodynamic parameters by impedance cardiography (ICG) in rats with MCT-induced PAH.

## Material and methods

### Animal preparation and experimental protocol

The study was conducted in the Biophysics Laboratory (Faculty of Medicine, Mersin University, Mersin, Turkey) between March and May in 2013. All experiments and protocols were performed according to the guidelines of the European Convention for the Protection of Vertebrate Animals used for Experimental and other Scientific Purposes (15) and approved by the Medical Faculty Experimentation Ethics Committee of Mersin University. Seventy male Wistar-Albino rats (12 week old; 220-320 g) were purchased from Clinical and Experimental Research Laboratory of Mersin University. They were housed under standard conditions (12 h light/dark cycle, 22 ± 2°C, relative humidity 50%-70%) and provided access to standard rat nutrients and purified drinking water *ad libitum*. After one week of acclimatization, rats were assigned randomly into the control group and four experimental groups – group treated only with MCT (PAH), PAH group treated with bosentan (PBOS; 300 mg/kg/d), PAH group treated with sildenafil (PSIL; 100 mg/kg/d), and PAH group treated with bosentan and sildenafil (PCOM; 300 mg/kg/d of bosentan and 100 mg/kg/d of sildenafil), each containing 14 animals. For induction of PAH, MCT dissolved in 1 N HCl (pH adjusted to 7.4 with 1 N NaOH; Sigma-Aldrich, Interlab Corporation, Istanbul, Turkey) was administered as a single subcutaneous injection of 60 mg/kg in a volume of 3 mL/kg after baseline measurement, while control rats received an equal volume of saline. Depending on the induction of PAH that was confirmed by echocardiography, treatments with PBOS, PSIL, or combined therapy were initiated 4 weeks after MCT injections and designated doses were given daily for 3 weeks in a volume of 2 mL by oral gavage. Control rats were administered the same amount of saline. No additional treatments were given to PAH rats. All rats were kept alive for 7 weeks and body weight was measured once weekly, while length (mouth to tail) was measured at the baseline, 4th, and 7th week.

### Indirect determination of blood pressure

Systolic (SBP) and mean arterial blood pressure (MABP) values were measured by tail-cuff method using a non-invasive indirect blood pressure system (MAY NIBP-200A, Commat Ltd Inc., Ankara, Turkey), which is compatible with BIOPAC MP100 Data Acquisition System (Biopac System Inc., Santa Barbara, CA, USA). The rats were first placed in an acrylic chamber (MAY Tail Heating-B, Commat Ltd Inc.) at 37°C for 20-30 min with an acrylic restrainer and then transferred to a standard setup with tail cuff and infrared sensor (RXTCUFSENSOR11, Biopac System Inc.), which is compatible with NIBP-200A. The cuff with infrared sensor was placed on the rat’s tail immediately after heating. SBP and MABP values were recorded with BIOPAC Acknowledge Software (V3.5.7, Biopac System Inc.) and the average value of at least five consecutive readings was obtained for each rat. Rats had been allowed to accustom to this procedure for 7 days before experiments were performed. All measurements were performed by the same person, who was blinded to the purpose of the study. SBP and MABP measurements were performed at the baseline, 4th, and 7th week.

### Transthoracic echocardiography (TTE)

**Figure 1:**
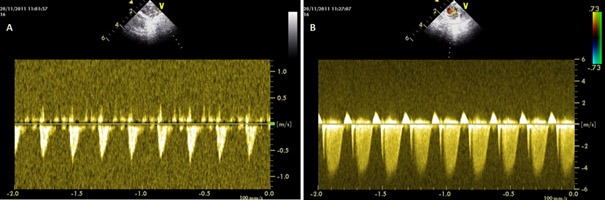
Transthoracic echocardiography (TTE) recordings in a rat with pulmonary arterial hypertension (PAH) obtained by Vivid I Cardiovascular Ultrasound System equipped with a 10 MHz probe. (**A**) An image of pulmonary arterial acceleration time (PAAT) and (**B**) An image of tricuspid valve regurgitation (TR).

RV function was determined by TTE using Vivid I Cardiovascular Ultrasound System (S/N: 001651, General Electric, Tirat Convel, Israel) equipped with a 10 MHz probe. Before procedure, rats were sedated with ketamine (100 mg/kg; ip), their anterior and bilateral (right and left) thoracic regions were shaved, and they were placed in anterior position. Ultrasound gel was placed on the thorax to optimize visibility. Pulmonary artery acceleration time (PAAT; ms) and RV systolic pressure from tricuspid valve regurgitation (TR) were measured (Figures 1A and B, respectively). PAAT values were assessed from parasternal view by pulse-wave Doppler and TR values were assessed from a 4-chamber apical view by color flow and continuous-wave Doppler at the best view position of the mitral and tricuspid valves. Right ventricular systolic pressure (RVSP_ECHO_; mmHg) was calculated using Bernoulli’s equation (RVSP_ECHO_ = 4 × TR^2^). TTE measurements were performed at the baseline, 4th, and 7th week and were completed within 5 to 10 min per rat.

### Impedance cardiography

**Figure 2:**
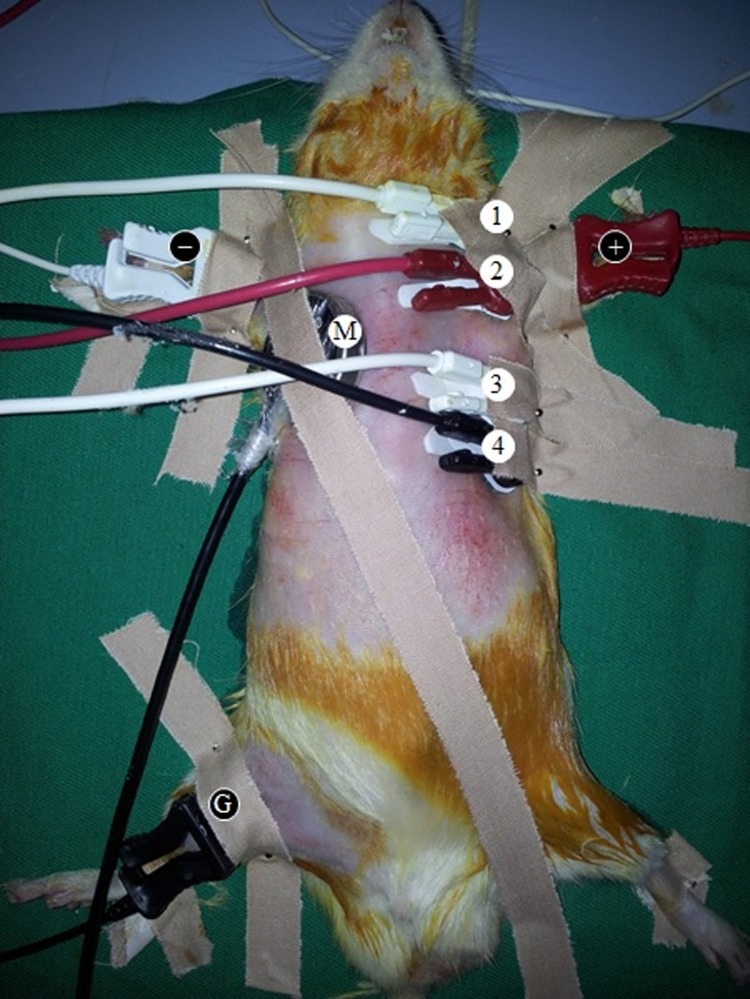
Impedance cardiography (ICG) recordings made by EBI100C module using surface electrode pairs (white dots 1 to 4). An electrical current was applied to the outer sensors (white dots 1 and 4) and voltage changes across the thorax were detected by the inner electrodes (white dots 2 and 3). Three standard electrocardiogram (ECG) electrodes were placed in Lead I configuration to record ECG signals (black dots +, –, G [ground]) and the signals were recorded by an ECG100C module. Heart sounds were recorded using DA100C module with a physiological microphone (white dot M).

**Figure 3:**
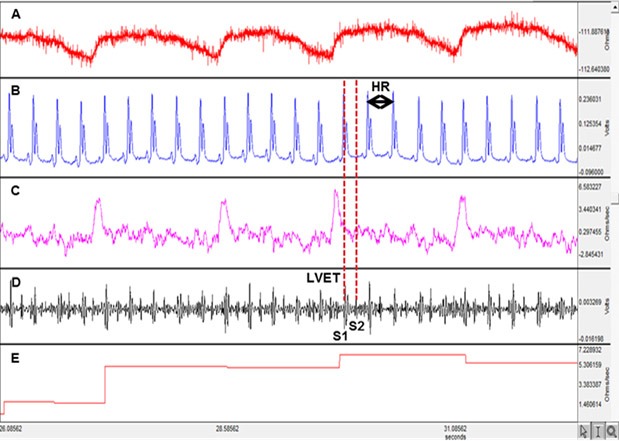
Simultaneous recordings of impedance cardiography (ICG), electrocardiogram (ECG), and heart sounds in a rat. (**A**) *Z*_0_ (steady state basal impedance). (**B**) ECG. Heart rate (HR) was detected by R-R interval detection method in ECG. (**C**) Rate of impedance change (d*Z*/d*t*). (**D**) Heart sounds. Left ventricular ejection time (LVET) was determined as the duration of electro-mechanical systole. Thus, LVET was measured as the time interval between the first (S1) and second (S2) heart sound using the heart-sounds trigger pulses. (**E**) Maximum rate of impedance change (d*Z*_max_/d*t*).

ICG recordings were performed by EBI100C module in BIOPAC MP 100 Acquisition System (Biopac System Inc.) in the same way as in our previous study (16). Immediately after TTE measurements, rats were placed in supine position and the left part of the neck was shaved. Four pairs of electrodes (Paired EL500 Electrodes, Biopac System Inc.) were placed on the left side of the neck and the thorax (Figure 2), and electrical current (70 kHz, 2.5 mA) was applied through the outer electrodes (the highest electrode on the neck and the lowest electrode on the thorax), while the inner electrodes measured the pulsatile changes in voltage. The voltage changes through the thorax reflect pulsatile alterations in thorax impedance and indicate stroke volume (SV; mL/beat). SV was calculated using the formula previously described by Sramek and Bernstein (17), VEPT × LVET × ((dZ/dt)_max_ / Z_o_), where LVET (sec) is the left ventricular ejection time, dZ/dt_max_ (ohm/s) the maximum rate of change in impedance, and Z_0_ (ohm) the base impedance. VEPT (mL) is the volume of electrically participating tissue, and was calculated from L^3^/4.25 ratio, where L (cm) is the distance between the inner electrodes (18). Cardiac output (CO; L/min) is the product of heart rate (HR; beats/min) and SV. To record HR, three standard ECG electrodes were placed in Lead I configuration (Figures 2 and 3) and ECG signals were recorded by an ECG100C module. Besides ECG, heart sounds were also recorded by DA100C module with a physiological microphone (TSD108, Biopac System Inc.) (Figure 2), and the interval between the first (S1) and the second (S2) heart sound was represented by LVET (Figure 3). Body surface area (BSA; m^2^) was calculated from rat’s length and weight using the Vallois formula (19), and it was used for the index calculations. Stroke volume index (SI; mL/beat/m^2^) and cardiac index (CI; L/min/m^2^) reflect the contractility of the heart and represent the ratios of SV/BSA and CO/BSA, respectively. Thoracic fluid content index (TFCI; 1/kohm/m^2^), which represents total thorax conductivity, was computed using the formula (1000/Zo)/BSA. Velocity index (IC; s^-1^), also known as myocardial contractility index, reflects the peak velocity of blood flow in the aorta during systole, and was calculated using the formula (dZ/dt)_max_/Z_o_ ratio. ECG, heart sounds, and ICG signals were continuously recorded for 60 sec (Figure 3) and measurements were performed at the baseline, 4th, and 7th week.

### Invasive determination of pulmonary arterial blood pressure (PABP_INV_)

Invasive pulmonary arterial blood pressure (PABP_INV_) measurements were performed at the 7th week. Before the procedure, rats were intubated with a 16-gauge plastic venflon, which was inserted directly into the trachea. Animals were subsequently attached to a mechanical ventilator (Harvard Apparatus 55-7059 inspira ASV ventilator, Holliston, MA, USA), ensuring normal breathing. Rats were ventilated under a peak pressure of 10 ± 2 cmH_2_O, a respiratory frequency of 60 ± 5 breath/min, and a FiO_2_ (fraction of inspired oxygen) value of 100%. After this, the mediastinum was opened by median sternotomy to locate the pulmonary artery. PABP_INV_ was measured by a 26-gauge plastic venflon that was directly connected to a pressure transducer (Biopac System Inc.) and signals were continuously recorded.

### Statistical analysis

Data were tested for normality of distribution with Kolmogorov-Smirnov test. Analysis of variance models were used to compare the groups at the baseline, 4th, and 7th week. Since at the baseline no treatment was applied to any group and since at the 4th week the same treatment (MCT treatment) was applied to four groups except the control, *post hoc* Dunnett’s test was performed to compare all groups with the control. At the 7th week, all groups were compared with each other using one-way analysis of variance (ANOVA) followed by Tukey multiple comparison test. Data were expressed as mean ± standard deviation (SD). The level of statistical significance was set at 0.05. Statistical analysis was performed using STATA/MP 11 software (Stata Corp LP, College Station, TX, USA).

## Results

At the baseline, each group consisted of 14 rats. No rats died in the control and PBOS group, and approximately 3-3.5 weeks after the MCT injection, 4 rats died in PAH group, 5 in PSIL group, and 1 in PCOM group. Therefore, at the 4th and 7th week, control group had 14, PAH had 10, PBOS had 14, PSIL had 9, and PCOM had 13 rats.

### Body surface area, systolic arterial blood pressure, and mean arterial blood pressure results

No significant differences in BSA (mean ± standard deviation, 0.035 ± 0.0032 m^2^), SABP (101.70 ± 20.69 mm Hg), and MABP (84.41 ± 19.58 mm Hg) were found between the groups at any measurement point.

### Transthoracic echocardiography results

At the baseline, mean PAAT, TR, and RVSP_ECHO_ values were similar in all groups (Table 1). At the 4th week, in all groups TR and RVSP_ECHO_ were significantly higher and PAAT was significantly lower than in the control group. At the 7th week, RVSP_ECHO_ and TR were still significantly higher in all groups, except in PSIL group, than in the control group. In PSIL group, the values were also significantly lower than in PAH, PBOS, and PCOM groups and similar to the control group (*P* < 0.001) (Table 1). PAAT values in all groups were lower than in the control group. PAAT values in PBOS and PSIL groups were higher than in PAH group. Also, PAAT values in PSIL group were higher than in PCOM group.

**Table 1:** Transthoracic echocardiography parameters. The results are expressed as mean ± standard deviation of the mean. One-way ANOVA was used to compare the groups at baseline, 4th, and 7th week. No significant differences in parameters were found between the groups at the baseline. In this case, pair wise comparisons were not used*

Parameters	CN	PAH	PBOS	PSIL	PCOM	*P*
**Baseline**						
PAAT (ms)	25.6 ± 3.0	27.6 ± 1.8	24.8 ± 2.2	25.3 ± 1.7	24.8 ± 2.0	0.053
TR (ms^-1^)	1.40 ± 0.15	1.27 ± 0.11	1.35 ± 0.10	1.30 ± 0.07	1.33 ± 0.07	0.080
RVSP_ECHO_ (mmHg)	9.3 ± 1.5	6.7 ± 0.9	8.1 ± 1.4	6.8 ± 0.6	7.1 ± 0.7	0.054
**4th week**						
PAAT (ms)	25.0 ± 1.6	18.6 ± 2.5^†^	20.6 ± 2.6^†^	17.6 ± 1.1^†^	16.9 ± 1.4^†^	<0.001
TR (ms^-1^)	1.7 ± 0.08	3.0 ± 0.09^†^	3.2 ± 0.50^†^	3.1 ± 0.24^†^	3.3 ± 0.63^†^	<0.001
RVSP_ECHO_ (mmHg)	12.1 ± 0.9	35.7 ± 2.1^†^	41.3 ± 14.4^†^	39.0 ± 5.7^†^	45.2 ± 18.1^†^	<0.001
**7th week**						
PAAT (ms)	25.7 ± 1.1	14.8 ± 1.1^‡^	20.4 ± 1.3^‡,§^	22.5 ± 1.8^‡,§,¶^	17.9 ± 3.8^‡^	<0.001
TR (ms^-1^)	1.6 ± 0.15	3.6 ± 0.4^‡^	3.6 ± 0.4^‡^	2.3 ± 0.4^§,║,¶^	3.6 ± 1.3^‡^	<0.001
RVSP_ECHO_ (mmHg)	10.3 ± 2.2	53.8 ± 11.7^‡^	52.1 ± 12.2^‡^	22.3 ± 7.9^§,║,¶^	57.9 ± 36.3^‡^	<0.001

*Abbreviations: CN – control rats treated with saline; PAH – pulmonary arterial hypertension induced rats with monocrotaline; PBOS – PAH rats treated with bosentan; PSIL – PAH rats treated with sildenafil; PCOM – PAH rats treated with bosentan and sildenafil; PAAT – pulmonary artery acceleration time; TR – tricuspid valve regurgitation; RVSP_ECHO_ – right ventricular systolic pressure.

**†**Compared to CN (4th week).

‡Compared to CN (7th week).

§Compared to PAH.

║Compared to PBOS.

¶Compared to PCOM.

### Impedance cardiography results

At the baseline and 4th week, there were no significant differences in the all ICG parameters between control group and other groups (Table 2). At the 7th week, no significant differences between the groups were found in TFCI, SI, and IC. However, CI was significantly higher in the PSIL than in control group. Also, CI in PSIL group had the greatest value compared to other groups. HR (which was remarkably decreased) was lower in PCOM than in other groups, while in PAH it was higher than in other groups. However, a significant difference was observed only between PCOM and PAH.

**Table 2:** Impedance cardiographic parameters. The results are expressed as mean ± standard deviation of the mean. One-way ANOVA was used to compare each parameter between the groups at the baseline, 4th, and 7th week. If no significant differences in parameters were found, pair wise comparisons were not used*

Parameters	CN	PAH	PBOS	PSIL	PCOM	*P*
**Baseline**						
TFCI (L/kohm/m^2^)	338 ± 159	352 ± 166	274 ± 59	309 ± 65	322 ± 132	0.745
SI (mL/beat/m^2^)	0.7 ± 0.9	0.5 ± 0.2	0.5 ± 0.2	0.8 ± 0.3	1.2 ± 0.6	0.054
HR (beats/min)	336 ± 67	373 ± 50	337 ± 56	366 ± 42	310 ± 85	0.262
CI (L/min/m^2^)	0.25 ± 0.35	0.20 ± 0.07	0.17 ± 0.09	0.30 ± 0.09	0.33 ± 0.13	0.310
IC (s^-1^)	0.06 ± 0.05	0.03 ± 0.01	0.04 ± 0.01	0.04 ± 0.01	0.06 ± 0.03	0.314
**4th week**						
TFCI (L/kohm/m^2^)	261 ± 99	288 ± 64	307 ± 91	280 ± 55	295 ± 107	0.816
SI (mL/beat/m^2^)	0.93 ± 0.29	0.94 ± 0.48	1.14 ± 0.75	1.11 ± 0.62	0.92 ± 0.29	0.822
HR (beats/min)	346 ± 57	371 ± 45	320 ± 43	370 ± 46	366 ± 69	0.145
CI (L/min/m^2^)	0.31 ± 0.07	0.34 ± 0.16	0.35 ± 0.22	0.41 ± 0.24	0.33 ± 0.08	0.827
IC (s^-1^)	0.05 ± 0.01	0.04 ± 0.02	0.05 ± 0.03	0.04 ± 0.03	0.03 ± 0.01	0.278
**7th week**						
TFCI (L/kohm/m^2^)	256 ± 40	320 ± 61	295 ± 114	298 ± 42	301 ± 83	0.413
SI (mL/beat/m^2^)	0.65 ± 0.38	0.90 ± 0.06	0.89 ± 0.61	1.21 ± 0.71	1.14 ± 0.44	0.099
HR (beats/min)	277 ± 59	367 ± 32	287 ± 65	292 ± 44	261 ± 57^‡^	0.044
CI (L/min/m^2^)	0.17 ± 0.08	0.33 ± 0.03	0.23 ± 0.15	0.34 ± 0.18^†^	0.29 ± 0.11	0.024
IC (s^-1^)	0.05 ± 0.01	0.03 ± 0.00	0.05 ± 0.03	0.04 ± 0.02	0.03 ± 0.01	0.193

*Abbreviations: CN – control rats treated with saline; PAH – pulmonary arterial hypertension induced rats with monocrotaline; PBOS – PAH rats treated with bosentan; PSIL – PAH rats treated with sildenafil; PCOM – PAH rats treated with bosentan and sildenafil; TFCI – thoracic fluid content index; SI – stroke volume index; HR – heart rate; CI – cardiac index; IC – velocity index.

†Compared to CN.

‡Compared to PAH.

### Invasive pulmonary arterial blood pressure (PABP_INV_) measurements results

PABP_inv_ values in PAH (38.50 ± 19.07 mm Hg) and PBOS group (42.00 ± 7.17 mm Hg) were significantly higher than in the control group (18.25 ± 6.14 mm Hg) (*P* < 0.05). No other significant differences in PABP_inv_ were found between the groups (PSIL – 32.1 ± 15.6 and PCOM – 30.9 ± 19.9 mm Hg).

## Discussion

Our study found that PAH did not have an effect on LV function in rats, while other studies (2,3,8,20,21) attributed the changes in cardiac function following administration of MCT to pulmonary hypertension.

The ICG is a non-invasive, continuous method with minimal technical requirements and no patient risk. The pulsatile impedance changes in the ICG directly reflect ascending aortic flow and, thus LV function. They are used to determine SV, which, when simultaneously measured with HR, can be used to calculate CO. In combination with heart sounds and ECG signals, ICG provides a continuous comprehensive hemodynamic profile for illness/treatment evaluations (16,18).

The MCT-induced PAH rat model has been widely used as an experimental model of PAH (22) and is the most similar animal model to PAH in humans (23). When injected into rats in a single subcutaneous or intraperitoneal administration, MCT undergoes hepatic transformation by cytochrome P450 monooxygenase in the liver to form MCT pyrrole, which causes muscular hypertrophy of the media of pulmonary vessels and leads to PAH and RV hypertrophy (24).

Our study found no significant difference in non-invasive blood pressure measurement value, TTE, and ICG between the groups at the baseline. Also, there were no differences in BSA, SABP, and MABP between the groups at the 4th and the 7th week of the study. Another MCT-induced model of PAH (25) showed no changes in mean arterial blood pressure, indicating normal LV function, but found higher RV systolic pressure. Indeed, we did not observe a change in hemodynamic parameters of LV only in MCT-treated rats. So, the impaired cardiac function of rats with PAH may depend only on RV dysfunction. However, Kögler et al (2) reported that the LV may also be involved since neurohormonal compensation of depressed cardiac function in PH and in certain types of congenital heart diseases affects both ventricles.

In this study, after 4 weeks TR values in PAH, PBOS, PSIL, and PCOM groups were significantly higher than in the control group. An increase in TR and a decrease in PAAT values were considered as sufficient evidence that PAH occurred in all MCT injected groups. Therefore, 4 weeks after treatment it was concluded that MCT rats had RV hypertension and hypertrophy unlike control rats.

On the other hand, TR and RVSP_ECHO_ values at the 7th week in the PSIL group were similar only to controls. In other words, improvement was observed only in PSIL group. Choudhary et al (26) reported that bosentan had no effect on RVSP or mass in normoxic animals. However, in our study, sildenafil plus bosentan did not affect RVSP_ECHO_ compared to sildenafil alone, which reduced this parameter. Such findings should be explained in future studies. Correia-Pinto et al (27) reported that at the sixth week MCT-treated rats had lower HR than MCT-treated rats at the fourth week, MCT-untreated rats at the fourth week, and MCT-untreated rats at the sixth week. Contrary to this, we observed that HR of MCT injected groups did not change at the 4th week, but that at the 7th week it was higher in PAH group and therapy groups, except PCOM group, than in controls. However, these increases were not significant. Mean HR in PCOM was significantly lower than in PAH group.

Our results indicated that sildenafil treatment deteriorated LV cardiac index. Clozel et al (28) showed that 300 mg/kg/d bosentan, 100 mg/kg/d sildenafil, or their combination for 4 weeks was the maximally effective dose in MCT-treated rats (28). In our study, bosentan and sildenafil were used in such maximally effective doses. Since our findings showed that sildenafil treatment deteriorated LV cardiac index, we recommend its use in lower doses. However, PABP_inv_ value in PSIL group was not significantly lower than in PAH group. Also, there was a significant difference at the 7th week in PABP_inv_ value between PSIL group and controls.

In a similar manner, TR and RVSP_ECHO_ values in PSIL group were similar to the control group. Clozel et al (28) reported a reduction in right ventricular hypertrophy in all MCT-induced PAH rat groups treated with bosentan, sildenafil, and combination therapy. In our study, PAAT values in the groups treated with sildenafil or bosentan increased significantly compared to PAH group. Also, although PAAT values in the combined therapy groups increased compared to rats with PAH, this increase was not significant. These results demonstrate that PAAT was the best ECHO parameter to determine the efficacy of treatment. However, it is surprising that after 21 days of treatment both sildenafil and bosentan did not lower PABP_inv,_ especially if we take into consideration that high doses of agents were used. Further studies should clarify such findings.

Echocardiography-based studies carried out in PAH patients suggested that one of the mechanisms contributing to LV dysfunction is ventricular interdependence and impaired LV filling (29). However, the timing when intrinsic LV myocardial dysfunction develops is not completely investigated.

Benoist et al (13) reported that in rats with MCT-induced PAH, electrical remodeling occurred not only in LV but also in RV, although in a lesser degree, resulting in increased electrical heterogeneity (13). This is not consistent with our study. Since rat size, LV function, and blood pressure values (BSA, SI, CI, IC, SABP, and MABP) did not differ significantly between the 5 groups, it seems that effects of PH are limited to changes in the RV.

There are a number of limitations of the present study that need to be addressed in future research. The first was that due to insufficient funding we did not use immunohistochemistry analysis. Thus, further studies should determine the ultrastructure of the two ventricles. In addition, besides the hemodynamic data, LV mass/whole heart mass or LV mass/tibial length could provide valuable information. Also, due to ethical and economic reasons, this study was designed to use the minimum number of rats necessary. Nevertheless, statistical analysis showed that the number of animals used in this study was sufficient to analyze the data correctly.

We can conclude that PAH did not have an effect on LV function of rats, or if it did, the effect was compensated by physiological processes. Our results also indicated that sildenafil treatment deteriorated the LV cardiac index.
